# Impact of a teaching strategy to promote evidence-based practice on nursing students’ knowledge and confidence in simulated clinical intervention choices

**DOI:** 10.1186/s12912-023-01540-1

**Published:** 2023-10-06

**Authors:** Itziar Estalella, Óscar Román, Theo Norbert Reichenberger, Amaia Maquibar

**Affiliations:** 1 Department of Nursing I, Faculty of Medicine and Nursing, University of the Basque Country UPV/EHU, B° Sarriena s/n, Leioa, Bizkaia, 48940 Spain; 2 University Library, University of the Basque Country UPV/EHU, B° Sarriena s/n, Leioa, Bizkaia, 48940 Spain

**Keywords:** Confidence-based marking, Education, evidence-based practice, Nursing, Students, nursing

## Abstract

**Background:**

Nurses self-efficacy, confidence and their competency for evidence-based practice have a relevant impact in the quality of care provided to patients. However, the implementation of evidence-based practice continues to be limited to date and the relationship between these elements has not been thoroughly understood. Thus, the aim of this study was to analyze the impact on confidence levels of a teaching strategy to promote evidence incorporation into clinical decisions made by student nurses in hypothetical scenarios. Besides, students’ satisfaction with the new teaching strategy was assessed.

**Methods:**

The teaching strategy was asynchronous, on-line and based on multiple-choice questionnaires related to decision making on an intensive care unit patient. Confidence levels were assessed by introducing the scoring tool confidence-based marking. Changes between pre- and post-tests in correct answers, confidence levels and expected-observed ranges of accuracy at each level of certainty were analyzed through non-parametric McNemar’s sign tests for paired-samples differences. To assess students’ satisfaction with the teaching strategy, a mixed-methods approach was followed. Descriptive statistical methods and Qualitative Content Analysis were followed respectively in order to analyze students’ satisfaction.

**Results:**

A total of 165 students completed the assignment, 101 answered the satisfaction survey and 7 participated in the interviews. Statistically significant better scoring and higher confidence levels were found in the post-intervention. Statistically significant differences in expected-observed ranges of accuracy were found for the three levels of certainty. Students were highly satisfied with the proposed task. In the qualitative analysis one category was elaborated which illustrated the students’ perceived added value of this new assignment.

**Conclusions:**

On-line teaching strategies based on clinical scenarios that focus on evidence-based decision-making have the potential to increase the confidence of nursing students. Additionally, interventions designed by teams incorporating clinical nurses, university librarians and academic nurses have the potential to bridge the evidence-practice gap in nursing education.

**Supplementary Information:**

The online version contains supplementary material available at 10.1186/s12912-023-01540-1.

## Background

Evidence-based practice (EBP) is a systematic problem-solving approach to the delivery of health care that improves quality and population health outcomes as well as reduces costs and empowers clinicians to fully engage in their role [1]. However, nurses still struggle to incorporate EBP into clinical practice [2, 3]. Some authors have suggested that low EBP competencies in terms of nurses’ EBP knowledge, skills and confidence are the main barrier to extended EBP implementation among registered nurses [3]. Supporting this same idea, other authors have found that despite showing a positive attitude towards EBP, and understanding its relevance for providing the best care, nurses often feel unprepared for its implementation [3, 4].

Incorporating new generations of nurses with better training in EBP into health systems could be an important part of the solution for this limited EBP. Consequently, EBP has been considered a core competence that student nurses should acquire, and it has been widely incorporated into the curricula of bachelor’s nursing studies worldwide. There are some recently published consensus-based sets of EBP core competencies and learning outcomes that detail what is required to become EBP competent [5, 6].

Given the complexity of EBP implementation, nursing educators face a huge challenge helping nursing students acquire this multi-layered competence, and associated skills, described above [7]. A recent study, based on data from six European countries [8], indicates that EBP teaching has been inconsistent and not sufficiently integrated, resulting in a low-moderate EBP competency rating among bachelor´s degree nurses. According to Aglen [9], one of the main challenges of teaching EBP is training students to recognize how research findings contribute to nursing practice. This challenge is not exclusive to EBP and is another strand of the unsolved theory-practice gap in nursing teaching [10]. Relating knowledge transfer to clinical problems and developing collaborative processes between nurse educators and clinical-based registered nurses have been suggested as ways to bridge the gaps in EBP teaching [9–11]. Furthermore, clinically integrated teaching methods have been found to improve not only EBP knowledge, but also attitudes [12]. However, reviews assessing the strategies for teaching EBP concluded that teaching focuses mainly on improving information literacy, critical appraisal of evidence and developing scientific inquiry, rather than on the translation of evidence into clinical practice [13–17].

Nurses’ confidence in their clinical decision-making is a key component of self-efficacy, and this has also been identified as essential for the implementation of EBP [12, 18]. According to Bandura’s social cognitive theory, self-efficacy refers to the belief in one’s own ability to successfully perform a behavior [19]. Although a strong correlation between self-efficacy and EBP has been observed, only a limited number of previous educational interventions have taken into consideration, or reported results on, the confidence of healthcare students. Some previous studies have described the use of self-reported confidence levels in university healthcare grades, in order to assess students’ awareness of the limits of their knowledge, discourage guessing, and, hypothetically, predict future clinical behavior [20–22]. Fewer studies have reported the effects of teaching interventions on students’ confidence level, and only one of them has addressed the relationship between an EBP approach and nurses’ confidence in clinical decision making [12, 23].

Online teaching/learning has become a relevant component of higher education over the past decade with a notable boost driven by the COVID-19 pandemic. Despite the importance student satisfaction has in relation to retention and academic performance, there is limited research analyzing student satisfaction with different online teaching strategies [24].

Therefore, the aim of this study was to analyze the impact on confidence levels of an online asynchronous teaching strategy to promote evidence incorporation into clinical decisions made by student nurses in hypothetical scenarios. Likewise, the study assessed students’ satisfaction with the teaching strategy proposed.

## Methods

### Teaching strategy description

The strategy assessed was an asynchronous formative assignment delivered online through our institution´s learning platform based on Moodle version 3.5 (Moodle Pty Ltd: West Perth, Australia). First, students were asked to answer a test that consisted of a multiple-choice questionnaire based on a clinical case. It included 17 questions related to nursing practice and decision-making on an intensive care unit (ICU) patient. Besides answering the multiple-choice questionnaire, students’ confidence in the correctness of the response given to each question was assessed by introducing Confidence-Based Marking (‘CBM’). This scoring tool developed by Gardner-Medwin requires students to state confidence at three levels: unsure (C1) when the expected probability of being right is < 67%, mid sure (C2) between 67 and 80%, or quite sure (C3) for a probability of > 80%. When grading, the tool rewards correct answers with a high confidence rate, while strongly penalizing students for incorrect answers rated as quite sure discouraging guessing and encouraging to state high levels of confidence only when there is a certainty on the answer being right [25].

Students received immediate feedback on each question when submitting the complete questionnaire through the platform. This feedback informed the students whether their answer was correct, their ‘CBM’ score according to the confidence level stated and a text to encourage them to review the recommended literature to better answer and support the given answer. Recommended reading ranged from recently published reviews to clinical guidelines and books. Finally, students were asked to answer the initial test again and were given a second feedback, where students were given the correct answer summarizing the evidence given and guidance on further resources or learning materials to go deeper into the topic (Supplementary Material 1).

The whole teaching strategy was designed by a team formed by two full-time academic nurses, a clinical nurse with extensive experience in the ICU and a university librarian. This team focused the teaching strategy on addressing the most relevant needs in relation to EBP nursing students had identified in a previous research [26]. To assess the feasibility, usefulness and appropriateness of the teaching strategy a pilot test with 28 voluntary students was carried on during the first semester of 2020. Given the positive results of the pilot intervention and the high satisfaction showed by participants, it was decided to implement it with the whole group of students in the following academic year.

### Study design

A pre-post quantitative study without a control group was conducted to assess the impact of the teaching strategy on students’ knowledge and confidence on their answers. To assess the students’ satisfaction with the teaching strategy, a mixed-methods sequential explanatory design approach was followed [27]. Quantitative data were collected through an online survey and qualitative data through in-depth individual interviews.

### Setting and participants

In Spain, where the present study took place, nursing studies underwent a profound restructuration of the whole curriculum because of the adaptation to the European Higher Education Area during the Bologna Process in 2008. This adaptation led to a shift in nurse education from more practice-oriented to more academic and research-oriented [28]. It was at that point that EBP was introduced into the regulation that describes the basic set of competences students must acquire to obtain the bachelor’s nursing degree, which requires the completion of 240 European Credit Transfer and Accumulation system in a 4-year degree at a university college [28]. This study was conducted at the public regional university that has a mean of 660 students enrolled distributed in the four years. The assessed teaching intervention was an activity presented to students during their clinical placements in a compulsory subject (Practicum V) of the fourth academic year (n = 180).

### Data collection

Data collection took place between January and June 2021. The assignment was asynchronous, meaning each student could decide when to access their institutional site in the Moodle platform to complete requested tasks during the months determined by the lecturers. All 180 students who were enrolled at the final-year at the time the data collection took place, were given the opportunity to take part in the teaching intervention. To assess students’ satisfaction with the assignment, a survey was launched in June 2021. All the students who had completed the assignment were invited *via* email to evaluate the teaching intervention by answering an anonymous online questionnaire and/or participating in individual interviews. Sample size for a 95% confidence level and a 5% margin error was estimated in 116 participants. In relation to qualitative data, the same two researchers, IE and AM, conducted all the interviews. Both are nurses with experience in qualitative research, had been lecturers of the participants in previous years, and were therefore known to the participants. Following a semi-structured interview guide, students were asked about their general perceptions on the training received during their university years, their perception of the training received in EBP in general, and of the new assignment in particular.

### Data analysis

Quantitative data analysis for both impact and satisfaction was conducted using IBM SPSS Statistics software version 26 (IBM Corp: Armonk, NY). Descriptive analysis was performed on the survey results and Mc Nemar’s non-parametric tests for related samples were used to analyze changes between pre- and post-tests. Values of p < 0.05 were considered statistically significant.

In relation to the qualitative data, interviews were transcribed verbatim and analyzed following Qualitative Content Analysis, as described by Graneheim and Lundman [29] with the aid of the Atlas.ti software version 9 (GmbH: Berlin, Germany). The analysis followed an inductive analysis approach and focused on manifest content. Initially meaning units, i.e. sections of each interview transcription that were related to the research question were identified and coded independently by the first and last author. The resulting list of codes was refined between both authors and all the authors discussed the relationship between the codes and possible groups until the elaboration of the final category described later in the results was elaborated. In relation to credibility, the different background and professional experience of the authors allowed triangulation during the process of data analysis and manuscript elaboration. In order to achieve dependability, we have tried to provide detailed information on how the analysis was carried out and have provided representative quotations from the interviews along with assigned codes to allow the reader assess the confirmability.

## Results

165 final year students completed the assignment, 101 of them filled out the satisfaction survey and 7 participated in the in-depth interviews.

### Pre and post-intervention tests comparison

Better scoring was found in the post-test with statistically significant differences for all questions (Table 1).

**Table 1:** Frequency of correct answers for each test question in the pre and post-tests (n = 165)

	Pre-test n (%)	Post-test n (%)	χ^2^_McN_ (p)
Question 1	145 (87.9)	164 (99.4)	17.05 (p < 0.001)
Question 2	149 (90.3)	165 (100)	14.06 (p < 0.001)
Question 3	142 (86.1)	164 (99.4)	18.35 (p < 0.001)
Question 4	99 (60.0)	164 (99.4)	61.13 (p < 0.001)
Question 5	70 (42.4)	164 (99.4)	92.01 (p < 0.001)
Question 6	124 (75.2)	162 (98.2)	34.22 (p < 0.001)
Question 7	122 (73.9)	150 (90.9)	15.85 (p < 0.001)
Question 8	131 (79.4)	163 (98.8)	26.70 (p < 0.001)
Question 9	149 (90.3)	161 (97.6)	6.05 (p = 0.014)
Question 10	101 (61.2)	158 (95.8)	53.15 (p < 0.001)
Question 11	141 (85.5)	164 (99.4)	21.04 (p < 0.001)
Question 12	146 (88.5)	164 (99.4)	14.45 (p < 0.001)
Question 13	157 (95.2)	164 (99.4)	4.00 (p = 0.039)
Question 14	126 (76.4)	164 (99.4)	34.22 (p < 0.001)
Question 15	121 (73.3)	161 (97.6)	36.21 (p < 0.001)
Question 16	87 (52.7)	161 (97.6)	72.01 (p < 0.001)
Question 17	122 (73.9)	162 (98.2)	33.06 (p < 0.001)

n: frequency; %: percentage; χ^2^_McN_: Non-parametric McNemar’s test for paired-samples differences; p: statistical significance level

In relation to confidence, there was greater variability in the stated levels in the pre-test. The percentage of students who were ‘quite sure’ (C3) in the pre-test was low for the majority of the questions (Rank 21–59%), and significantly higher in the post-test (Rank 86–98%). These changes resulted in statistically significant differences in pre- and post-test confidence levels in all questions (Table 2). Expected-observed ranges of accuracy at each level of certainty were analyzed finding statistically significant differences at pre and post-tests for the three levels of certainty. Thus, in the second attempt, the majority of students did not answer questions with insecurity (C1), and out of the few who chose this lowest level of confidence (n = 19), 16 got more right answers than expected for this level of confidence and 3 were in the expected range (χ2McN-B = 117,87; gl = 3; p < 0,001). Similarly, in the second attempt, the majority of participants did not answer questions with mid-confidence (C2) and the ones who chose this confidence level showed an accuracy above the range expected (χ2McN-B = 123,28; gl = 6; p < 0,001).

**Table 2:** Frequency of confidence levels stated for each question in the pre and post-tests (n = 165)

	Pre-test n (%)				Post-test n (%)			Sign difference (p)
	C1	C2	C3		C1	C2	C3	
Question 1	16 (9.7)	57 (34.5)	92 (55.8)		0 (0)	4 (2.4)	161 (97.6)	8.18 (p < 0.001)
Question 2	13 (7.9)	64 (38.8)	88 (53.3)		0 (0)	9 (5.5)	156 (94.5)	8.07 (p < 0.001)
Question 3	20 (12.1)	67 (40.6)	78 (47.3)		1 (0.6)	12 (7.3)	152 (92.1)	8.36 (p < 0.001)
Question 4	40 (24.2)	56 (33.9)	69 (41.8)		0 (0)	9 (5.5)	156 (94.5)	9.18 (p < 0.001)
Question 5	19 (11.5)	49 (29.7)	97 (58.8)		0 (0)	6 (3.6)	159 (96.4)	7.36 (p < 0.001)
Question 6	83 (50.3)	46 (27.9)	36 (21.8)		4 (2.4)	18 (10.9)	143 (86.7)	10.60 (p < 0.001)
Question 7	47 (28.5)	65 (39.4)	53 (32.1)		1 (0.6)	21 (12.7)	143 (86.7)	9.81 (p < 0.001)
Question 8	55 (33.3)	48 (29.1)	62 (37.6)		2 (1.2)	10 (6.1)	153 (92.7)	9.60 (p < 0.001)
Question 9	43 (26.1)	47 (28.5)	75 (45.5)		3 (1.8)	10 (6.1)	152 (92.1)	8.21 (p < 0.001)
Question 10	23 (13.9)	71 (43.0)	71 (43.0)		1 (0.6)	10 (6.1)	154 (93.3)	8.90 (p < 0.001)
Question 11	32 (19.4)	66 (40.0)	67 (40.6)		2 (1.2)	8 (4.8)	155 (93.9)	9.29 (p < 0.001)
Question 12	49 (29.7)	44 (26.7)	72 (43.6)		2 (1.2)	7 (4.2)	156 (94.5)	9.01 (p < 0.001)
Question 13	35 (21.2)	43 (26.1)	87 (52.7)		2 (1.2)	5 (3.0)	158 (95.8)	8.14 (p < 0.001)
Question 14	63 (38.2)	49 (29.7)	53 (32.1)		4 (2.4)	11 (6.7)	150 (90.9)	9.62 (p < 0.001)
Question 15	64 (38.8)	42 (25.5)	59 (35.8)		1 (0.6)	12 (7.3)	152 (92.1)	9.67 (p < 0.001)
Question 16	49 (29.7)	77 (46.7)	39 (23.6)		4 (2.4)	20 (12.1)	141 (85.5)	10.7 (p < 0.001)
Question 17	76 (46.1)	54 (32.7)	35 (21.2)		1 (0.6)	13 (7.9)	151 (91.5)	10.83 (p < 0.001)

n: frequency; %: percentage; Sign difference: Non-parametric sign test for paired-samples differences; p: statistical significance level. C1 = Unsure. C2 = Mid sure. C3 = Quite sure

### Students’ satisfaction with the task proposed

The 82% of students who filled out the survey were female and 73% were in the age range of 21–23 years. Descriptive analysis from the anonymous online survey shows high satisfaction with the assignment proposed. None of the participants in the survey considered the workload of the task to be too much burden, 66% considered it to be a reasonable burden, 28% little burden and 6% not a burden. Furthermore, 99% of participants stated that the feedback from the first attempt had been useful or more than useful to encourage them to review evidence given. In relation to the usefulness of the task to encourage them to search for research evidence the next time they faced a doubt during their clinical practice, 56% considered the assignment completely useful, 30% quite useful, 11% useful and 3% little useful. None of the respondents considered it useless. In relation to self-confidence, 93% of students stated the assignment had been useful, quite or completely useful to increase their self-confidence and supported offering this assignment in the coming years (Table 3).

**Table 3:** Students’ satisfaction with the task proposed. Survey questions and results (n = 101)

	%
The workload of accomplishing the task has been:	
It has not supposed a burden	6
Little burden	28
It has supposed a burden but it is reasonable	66
Too much burden	0
Do you think the test fits the clinical scenario of the critical patient?	
It doesn´t fit / Fits little	0
Fits regular	11
Fits quite a lot	45
Fits completely	44
Has the feedback from the first attempt been useful to review the evidence given?	
Useless / Little useful	1
Useful	18
Quite useful	36
Completely useful	45
Has the feedback from the second attempt been useful to understand?	
Useless / Little useful	1
Useful	7
Quite useful	47
Completely useful	46
Has the task been useful to encourage you to search for evidence when in doubt next time in clinical practice?	
Useless / Little useful	3
Useful	11
Quite useful	30
Completely useful	56
Has the task been useful to increase self-confidence now that you are going to start your professional career?	
Useless / Little useful	7
Useful	21
Quite useful	30
Completely useful	42

%: percentage

From the qualitative analysis, one category was elaborated: ‘The added value’. This category includes all the codes related to students’ perception of what this assignment had added to their previous training (Table 4).

**Table 4:** Students’ satisfaction with the task proposed. In-depth interviews (n = 7). Category emerged from the data analysis with quotations and related codes

Category	Code	Quotation
The added value	The assignment makes them aware of their knowledge	‘*I appreciate you had this initiative to do this type of assignment because I feel I have really learnt, that I have seriously addressed doubts I had or things I was not so sure about.*’ Student 3 *‘I thought I had super clear the answer and then when I saw the feedback I though “No way! I was wrong!” So yes, in that sense, I think you created the emotion, well, I don’t know how to call it, motivation.’* Student 2
	Assignment and spirit of inquiry	*‘Your task was really useful for me, there were some questions that really got me hooked so I read everything because I thought “How is it possible for me to not know this?”* [laughs] *so I started reading and yes, in my opinion, the questions you asked were very specific and they are dilemmas you can find in your nursing practice… and thanks to the Practicum I now know that.’* Student 2 *‘…and I remembered the task. Seeking for answers, inquiring, making us think… in the end it gives you knowledge, knowledge that you can apply to your experience, and yesterday for example* [at the hospital] *I recalled this task from the Practicum*.’ Student 1
	Positively assess feedback	*‘To be honest I was positively surprised with the feedback because I thought it was going to be just “wrong, wrong, right” and the truth is you worked hard to specify “this is wrong, look here, read this” so I think the feedback was great, I mean., I was really surprised. When things are correctly done, they are correctly done, and I think this was. I loved the idea that you had and I believe it’s been very useful.’* Student 3
	Encourage to seek information	*‘The assignment is just perfect but maybe it would be good that, besides having the access to the documentation, the student was encouraged to seek for the information, to learn how to look for that documentation.’* Student 5
	Positive that it is based on a clinical case	*‘Yeah, I think case studies “on the paper” are quite good especially before you face real clinical cases, at the end, you get a bit of an idea, it makes you think.’* Student 5 *‘Yes, the truth is these questions focused on clinical cases are very interesting to reinforce clinical practice because they are situations that you really face in your nursing practice, well, of course, we don’t have placements in all the different units. For example, I haven´t been in an ICU and your case was based on an ICU patient, so it’s been useful to reinforce clinical practice and learn about services where we don´t all have the chance to stay*.’ Student 6
	Worthwhile workload	*‘The workload was reasonable. I mean, nobody likes doing assignments, obviously, because when you get home from clinical practice you’re tired, you have other things to do also. But in relation with usefulness, taking into account usefulness by time spent … this practical case is more useful than other assignments’* Student 4
	Scale-up	*‘I think the task of the ICU is a good idea, but it also seems like a good idea to contextualize it to the different subjects, for example, in a cancer patient or a hip fracture because in the end that is what we are going to see. So it is important and I find it very interesting to learn for real work.’* Student 6

In line with the survey results, interviewees expressed high satisfaction with the proposed assignment. This category is about which elements students valued more positively and what made them consider this new teaching strategy as a better option.

Participants highlighted as positive characteristics of this assignment, in comparison with previous ones, the use of the ‘CBM’ tool and the received feedback. These two elements were key for raising awareness among students about their knowledge and triggering them to consult the proposed materials; although some participants would have preferred to be encouraged to search for the evidence themselves instead of being given the documents.

The participants valued the ease with which the questions derived from the clinical case could be linked to their experiences during clinical placements highly. Students who had worked in an ICU unit during their placements valued how well it was aligned with current practice, and those who had never worked in such a unit valued how it helped to prepare them for possible work there in the future.

Consequently, even though the workload was considered higher, they stated it was ‘doable’ and worthwhile. There was a consistent recommendation to offer this activity again to the next generation of student nurses. Some of the students suggested the scale-up of the intervention to other subjects/years and pointed out its potential as an evaluation tool for assessing acquired knowledge during clinical placements.

## Discussion

The aim of this study was to analyze the impact on confidence levels of a teaching strategy to promote evidence incorporation into clinical decisions made by student nurses in hypothetical scenarios. Overall, the strategy resulted in better scoring and higher confidence levels. Moreover, the students reported high satisfaction with the task proposed. They specially appreciated the strong link between the assignment and current clinical practice and the received feedback.

A meta-analysis aimed at comparing EBP teaching with traditional teaching mode, found EBP teaching was more effective in developing critical thinking among nursing students [30]. Our results showed a statistically significant higher confidence in the answers to questions related to clinical decision-making after evidence was consulted and analyzed by students. Thus, our findings suggest that EBP teaching strategies that guide students through steps 4 and 5, apply and reflect, of the EBP process can not only improve critical thinking, but also increase student nurses’ confidence in clinical decision making in hypothetical clinical scenarios [6, 30]. In this study, student nurses’ confidence in providing the correct care to patients increased by knowing that their clinical decision was supported by research evidence. This effect might be enhanced among student and junior nurses who still lack the clinical experience to support decisions made. Therefore, activities and assignments similar to the one described in this article, which emphasize the application of research evidence to clinical decision making, might increase students’ self-efficacy and better prepare them for the challenges they will face when integrating into the healthcare system [18].

Kim et al. [12] found self-confidence in clinical decision-making to be a significant predictor variable for the current and future use of EBP. Based on our findings we hypothesize self-confidence and EBP could be intertwined in the sense that greater use of evidence increases self-confidence and therefore those with higher confidence are more aware of the relevance of EBP use and will use it more. More research studies analyzing the relationship between confidence and EBP are needed to better understand this relationship.

According to the qualitative results, the high levels of satisfaction with the new assignment might be due to the ability of this teaching strategy to close the theory-practice gap, which had been pointed out previously as a barrier to students understanding the incorporation of evidence into their practice [10, 26]. The theory-practice gap in nursing teaching remains an unsolved issue that has its origins in the shift from hospital-based training to university-led education [31]. In the assessed teaching strategy answering the questions required students to apply an EBP framework by integrating theory learnt in classes, alongside relevant research evidence, in a clinical context. Furthermore, the received feedback stressed the implications of the decisions made on the patient, in an effort to make students aware of the relevance of evidence for nursing practice, as suggested by previous studies [9]. In the case of the assessed intervention, having in the team a clinical nurse with expertise in an ICU allowed the clinical scenario and the feedback to be updated to current nursing practice and facilitated the formulation of focused clinical questions that students could easily relate to their experience as student nurses. Thus, our findings support the recommendation of developing collaborative processes between clinical nurses and nurse educators to close the theory-practice gap in EBP teaching [7, 10].

Moreover, this intervention took into consideration institutional strengths identified in the literature, specifically the EBP knowledge and skills of university librarians, by including one in the work team that designed and implemented the intervention [32], as well as some key elements previously identified as effective in the literature for Internet-based learning, such as interactivity, repetition and feedback [33].

### Implications for research

There is a need for further evaluations of the impact educational interventions designed and implemented by clinical nurses alongside academic nurses might have in bridging the evidence-practice gap. The development of clinical scenarios from which questions related to nursing interventions are derived might be a feasible, simple and easy way to begin this collaboration. Similarly, future interventions for EBP teaching that incorporate university librarians should be described and evaluated in relation to the potential benefits this inter professional collaboration might add to such interventions.

Further analyses of the relationship between EBP teaching and other relevant skills and competences such as critical thinking, confidence and clinical decision making could reveal relevant interactions that nurse educators might consider for a better professional development of student nurses.

### Limitations

The main limitation of the present study is that the impact was analyzed through a pre-post study without a control group. Other study designs with greater statistical power that implied a control group were discarded due to ethical issues related with making differences in the training offered to students who belong to the same academic program and the high risk of contamination between both groups.

Another limitation is the small number of participants. In relation to the impact of the teaching intervention, because of the small sample, results should be interpreted with caution until repeated measurements over the years confirm or reject the preliminary results presented in this paper. Similarly, future studies should assess whether the changes seen in decision making in hypothetical scenarios translate into changes in clinical practice. In relation to students’ satisfaction, even though the response rate to the satisfaction questionnaire was good, 67%, the sample was not representative of the group in terms of size and the number of interviews was not enough to ensure data saturation was achieved. Thus, there might be other reasons besides the ones identified in this study for the high satisfaction found.

## Conclusion

Online teaching strategies based on clinical scenarios that focus on evidence-based decision-making have the potential to increase the confidence of nursing students. To achieve this, it is essential that the feedback provided to the students highlights the relevance of basing clinical decisions on best scientific evidence. Additionally, interventions designed by teams incorporating clinical nurses, university librarians and academic nurses have the potential to bridge the evidence-practice gap in nursing education.

### Electronic supplementary material

Below is the link to the electronic supplementary material.

Supplementary Material 1: Assignment overview: Example of three of the questions and feedbacks used in the assignment with a brief summary of the fictitious-based clinical scenario presented to the students. Students had also available the data that would have been collected in the admission assessment following Gordon´s functional health patterns. All data were made up by the authors.

## Data Availability

The datasets used and/or analyzed during the current study are available from the corresponding author on reasonable request.

## Abbreviations

CBM: Confidence-based marking
EBP: Evidence-based practice
ICU: Intensive care unit
