# Supplementary material for: Impact of a teaching strategy to promote evidence-based practice on nursing students’ knowledge and confidence in simulated clinical intervention choices
Source: BMC Nurs. 2023 Oct 6;22:361. doi: 10.1186/s12912-023-01540-1 (PMC10559451; doi:10.1186/s12912-023-01540-1)
Supplement: Supplementary file 1 — Supplementary Material 1: Assignment overview: Example of three of the questions and feedbacks used in the assignment with a brief summary of the fictitious-based clinical scenario presented to the students. Students had also available the data that would have been collected in the admission assessment following Gordon´s functional health patterns. All data were made up by the authors. [file 12912_2023_1540_MOESM1_ESM.docx]

| A 69-year-old man admitted to the Intensive Care Unit (ICU) from the digestive ward. He underwent surgery a week ago for hepatectomy but has progressively become unwell during the last 2 hours prior to admission in our unit. | | |
| --- | --- | --- |
| Question 2 | Since the patient has just arrived to the unit, risk of developing a pressure ulcer needs to be reassessed. To do so, you will use:  a) The Braden risk assessment scale, which is the only validated clinical scale to assess the risk of developing a pressure ulcer, and therefore the one included in the hospital care management tool.  b) Although I know that there are several pressure ulcer risk assessment tools translated into Spanish and validated, e.g., Braden, Norton or EMINA, as I am working at Osakidetza, I will use the Braden scale, which is the one included in the hospital care management tool.  c) Since the patient has just been admitted to the ICU, it is not necessary to assess the risk of PU on admission; it is better to do it later but always within the first 8 to 72 hours. | |
|  | Feedback 1 | a) Unfortunately, your answer is not correct! Although it is the scale included in the hospital care management tool, it is not the only one. To learn more about which scales you could use at this or other health services we invite you to read the resources corresponding to this question included in the *‘Recommended literature’* section.  b) That's right! Among the existing scales, Osakidetza has chosen the Braden scale to include it as part of its care management tool. However, it is important for you to know that there are other validated scales you could use in clinical practice, e.g., Norton or EMINA, and that are also recommended by the Spanish National Advisory Group for the Study of Pressure Ulcers and Chronic Wounds (GNEAUPP). If you are interested in going deeper into this topic, we encourage you to read the GNEAUPP Technical Document *‘Scales and instruments for assessing the risk of developing pressure ulcers’*, available in the section *‘Recommended literature’* for this question 2.  c) Unfortunately, your answer is not correct! It’s our fault if a person we are taking care of develops pressure ulcers. To learn the basics about pressure ulcer prevention and which scales you could use in this and other services we invite you to read the resources included in the section ‘*Recommended literature’* for questions 2 and 3. Don't procrastinate, this is very important! |
|  | Feedback 2 | a) Unfortunately, your answer is not correct! Although it is the scale included in the hospital care management tool it is not the only one. In addition to Braden Scale, there are other validated scales you could use in clinical practice, e.g., Norton or EMINA, and that are also recommended by the Spanish National Advisory Group for the Study of Pressure Ulcers and Chronic Wounds (GNEAUPP). We strongly encourage you to read the GNEAUPP Technical Document *‘Scales and instruments for assessing the risk of developing pressure ulcers’*, available in the section *‘Recommended literature’* for this question. Additionally, it is important not to rely patient’s individualized care plan on the risk score alone, but use a comprehensive approach to risk assessment to identify pressure ulcer risk factors. Keep in mind that you are taking care of a critically ill and perioperative patient, which implies many other specific factors besides the one in the scale should be taken into account.  b) That's right! Among the existing scales, Osakidetza has chosen the Braden scale to include it as part of its care management tool. However, it is important for you to know that there are other validated scales you could use in clinical practice, e.g., Norton or EMINA, that are also recommended by the Spanish National Advisory Group for the Study of Pressure Ulcers and Chronic Wounds (GNEAUPP). Remember also the importance of using scales that have been translated and validated in our context whenever possible. Still, do not rely patient’s individualized care plan on the risk score alone, but use a comprehensive approach to risk assessment to identify pressure ulcer risk factors. Keep in mind that you are taking care of a critically ill and perioperative patient, which implies many other specific factors besides the one in the scale should be taken into account.  c) Unfortunately, your answer is not correct! It’s our fault if a person we are taking care of develops pressure ulcers. Among the existing scales, Osakidetza has chosen the Braden scale to include it as part of its care management tool. Still, do not rely patient’s individualized care plan on the risk score alone, but use a comprehensive approach to risk assessment to identify pressure ulcer risk factors. Keep in mind that you are taking care of a critically ill and perioperative patient, which implies many other specific factors besides the one in the scale should be taken into account. Assessment must be done on admission within 8 hours. Only in these exceptions is it not mandatory: ‘*women admitted to obstetrics for delivery; persons admitted to a mental health unit, except patients admitted to psychogeriatrics or patients with eating disorders; patients admitted for hospital stays of less than 24 hours and assessment of PU risk can be postponed for patients undergoing surgery until after surgery*’ (Osakidetza guideline for pressure ulcer prevention, 2017, page 20). |
| Question 6 | An hour later, his daughter calls to let you know he is in pain. Using the visual analogue scale you conclude the score is seven. The patient carries a central venous catheter and an epidural catheter. Treatment prescribed includes Morphine 4 mg/6 hours and Ropivacaine 4 mg/4 hours in case of pain. What is the route of administration?  a) Both medications can be administered by intravenous route.  b) Both medications can be administered into the epidural space.  c) Administer the prescribed Morphine intravenously and Ropivacaine epidurally. | |
|  | Feedback 1 | a) & b) Unfortunatly your answer is not correct! Medication errors (dose calculation, route of administration, etc.) are one of the most frequent causes of iatrogenesis, compromising patient safety. Do not be discouraged, but please review the resources included in the section *‘Recommended literature’* for this question.  c) Very good, that is correct! Medication errors (dose calculation, route of administration, etc.) are one of the most frequent causes of iatrogenesis compromising patient safety. You have just avoided one of these errors by properly administering the prescribed medication, well done! Ropivacaine poisoning is most often due to the mistake of injecting the drug intravenously. Morphine can be administered by epidural route but should not exceed 10mg/24h. In both cases, serious adverse effects may occur, mainly affecting the central nervous and cardiovascular systems. If you want to learn more about the use of both substances in analgesia or if you want review the most common drugs used in critical care please see the *"Recommended literature"* section for question 6. |
|  | Feedback 2 | a) You have just compromise your patient safety! Ropivacaine poisoning is most often due to the mistake of injecting the drug intravenously, which could lead to serious adverse effects affecting mainly the central nervous system (CNS), e.g. seizures, and less frequently, but more severely, cardiovascular alterations, e.g. Hypotension, arrhythmias and cardiac arrest. Please see the provided book '*Human Pharmacology'*, section 5: *'Adverse Reactions'*, page 296. You can also consult Ropivacaine data sheet from the Spanish Agency of Medicines and Health Products (AEMPS) (section 4.8). We also encourage you to read the attached review by El-Boghdadly et al. (2018), where the CNS and cardiovascular adverse effects are highlighted as the most relevant and reference is made to the administration of Lipid Emulsion for the rescue of intoxication (p. 41).  b) You have just compromise your patient safety! Morphine can be administered into the epidural space but should not exceed 10mg/24h, as you can read on page 2 of the AEMPS data sheet. The most pernicious adverse effect could be respiratory depression of the patient (AEMPS data sheet, p. 7). We recommend reading the article on a recent clinical case described by Akkerman et al. (2019) included in the section.  c) Very good, that is correct! You have just avoided one of these errors by properly administering the prescribed medication, well done! Ropivacaine poisoning is most often due to the mistake of injecting the drug intravenously, which could lead to serious adverse effects affecting mainly the central nervous system (CNS), e.g. seizures, and less frequently, but more severely, cardiovascular alterations, e.g. hypotension, arrhythmias and cardiac arrest. Please see the book '*Human Pharmacology'* provided, section 5: *'Adverse Reactions'*, page 296. You can also consult Ropivacaine data sheet from the Spanish Agency of Medicines and Health Products (AEMPS) (section 4.8). We also encourage you to read the attached review by El-Boghdadly et al. (2018), where the CNS and cardiovascular adverse effects are highlighted as the most relevant and reference is made to the administration of Lipid Emulsion for the rescue of intoxication (p. 41). Morphine can be administered by epidural route but should not exceed 10mg/24h, as you can read on page 2 of the AEMPS data sheet. The most pernicious adverse effect could be respiratory depression of the patient (AEMPS data sheet, p. 7). We recommend reading the article on a recent clinical case described by Akkerman et al. (2019) included in the section. |
| Question 10 | Suddenly, his daughter calls you urgently, very nervous, to tell you that she thinks her father is getting worse. Just a few minutes before, you have been asked to prepare your patient for a scan test because, as his daughter senses, things are not going well. The daughter asks you insistently what is going on, she wants information, but you are in a hurry organizing what is needed for the test as soon as possible. What would you do?  a) The main priority now is preparing the patient for the scan procedure; the family can wait until this is done.  b) I would kindly invite her to leave the room and to seek for the doctor in charge to talk to her.  c) As far as possible, I would try to attend to her needs, help her understand the situation and explain what’s going on from an empathetic point of view. | |
|  | Feedback 1 | a) This is not the best option. In a situation like this where there is no imminent life threat, the patient's family, unless the patient has indicated otherwise, is your secondary patient according to some authors, and therefore you must also take care of them. Acquiring tools to establish therapeutic communications with patients and their families is essential if you want to be a good nurse. We encourage you to review question 10 *'Recommended literature'* section for this purpose.  b) This is not the best option. Establishing therapeutic communication with patients is an essential part of nursing and the patient's family is your secondary patient according to some authors and unless the patient has stated otherwise. We encourage you to review question 10 *'Recommended literature'* section that will help you acquire tools to establish therapeutic communication and become a better professional.  c) Yes! Unless stated otherwise by the patient, your patient's family is your secondary patient according to some authors and, therefore, you should attend to their needs by trying to establish therapeutic communication whenever there is a possibility. If you want to learn more about how to establish therapeutic communication with your patients and their families we invite you to review the *'Recommended literature'* section for this question. |
|  | Feedback 2 | a) This is not the best option. In a situation like this where there is no imminent life threat, the patient's family, unless the patient has indicated otherwise, is your secondary patient, as indicated in Valverde-Gefaell's *'Therapeutic Communication in Nursing'* guide, and therefore you should also pay attention to her. You may not be able to dedicate much time to her in these circumstances, but you can make her feel that you understand her concern, give her a minimum of information and state that you will dedicate the necessary time to clear up all her doubts and give her the opportunity to express her fears and concerns as soon as possible. Establishing therapeutic communications with patients and their families is essential if you want to be a good professional. If you have not had the time to do so, we encourage you to read the guide included in the folder, which we believe provides very useful information in this regard.  b) This is not the best option. Establishing therapeutic communication with patients is an essential part of nursing and the patient's family is your secondary patient as indicated in Valverde-Gefaell's *'Therapeutic Communication in Nursing'* guide, and therefore you should pay attention to her. You may not be able to dedicate much time to her in these circumstances. But you can make her feel that you understand his concern, give her a minimum of information and express that you will dedicate the necessary time to clear up all her doubts and give her the opportunity to express his fears and concerns as soon as possible. Establishing therapeutic communications with patients and their families is essential if you want to be a good professional. If you have not had the time to do so, we encourage you to read the guide included in the folder, which we believe provides very useful information in this regard.  c) Yes! Your patient's family is your secondary patient according to Valverde-Gefaell's *'Therapeutic Communication in Nursing'* guide, and therefore you should attend to their needs by trying to establish therapeutic communication whenever there is a possibility. In this case you may not be able to spend much time with them, but you can make them feel that you understand their concerns, give them a minimum of information and express that as soon as possible you will dedicate the necessary time to clear all their doubts and give them the opportunity to express their fears and concerns. |

ICU: Intensive Care Unit; Osakidetza: Basque Health Service; GNEAUPP: Spanish National Advisory Group for the Study of Pressure Ulcers and Chronic Wounds; AEMPS: Spanish Agency of Medicines and Health Products; CNS: Central Nervous System
